# MiR-378 exaggerates angiogenesis and bone erosion in collagen-induced arthritis mice by regulating endoplasmic reticulum stress

**DOI:** 10.1038/s41419-024-07193-5

**Published:** 2024-12-18

**Authors:** Zhengmeng Yang, Nan Hou, Wenxiang Cheng, Xuan Lu, Ming Wang, Shanshan Bai, Yuejun Lin, Yaofeng Wang, Sien Lin, Peng Zhang, Micky D. Tortorella, Lu Feng, Gang Li

**Affiliations:** 1 Centre for Regenerative Medicine and Health, Hong Kong Institute of Science & Innovation, Chinese Academy of Sciences, Hong Kong SAR, PR China; 2 Stem Cells and Regenerative Medicine Laboratory, Li Ka Shing Institute of Health Sciences, The Chinese University of Hong Kong, Prince of Wales Hospital, Shatin, Hong Kong SAR PR China; 3 Musculoskeletal Research Laboratory, Department of Orthopaedics & Traumatology, Faculty of Medicine, The Chinese University of Hong Kong, Prince of Wales Hospital, Shatin, Hong Kong SAR PR China; 4 Institute of Translation and Medical Research and Development Center, Shenzhen Institute of Advanced Technology, Chinese Academy of Sciences, Shenzhen, PR China; 5 The CUHK-ACC Space Medicine Centre on Health Maintenance of Musculoskeletal System, The Chinese University of Hong Kong Shenzhen Research Institute, Shenzhen, PR China

**Keywords:** Rheumatoid arthritis, miRNAs

## Abstract

Rheumatoid arthritis (RA) is a chronic autoimmune disorder marked by pain, inflammation, and discomfort in the synovial joints. It is critical to understand the pathological mechanisms of RA progression. MicroRNA-378 (miR-378) is highly expressed in the synovium of RA patients and positively correlated with disease severity, but its function and underlying mechanisms remain poorly understood. In this study, miR-378 transgenic (miR-378^high^) mice were used to construct the collagen-induced arthritis (CIA) model for exploring the role of miR-378 in RA development. miR-378^high^ CIA mice showed accelerated RA development, as evidenced by exaggerated joint swelling and bone structural deformities. More severe endoplasmic reticulum (ER) stress and the consequent angiogenesis and osteoclastogenesis were also activated in the synovial tissue and calcaneus, respectively, in the miR-378^high^ group, suggesting that ER plays a significant role in miR-378-mediated RA pathogenesis. Upon in vitro RA induction, fibroblast-like synoviocytes (FLSs) isolated from miR-378^high^ mice showed a higher expression level of ER stress markers. The conditioned medium (CM) from RA-FLSs of miR-378^high^ mice stimulated more intensive angiogenesis and osteoclastogenesis. The ER stress-related protein Crebrf was identified as a downstream target of miR-378. Crebrf knockdown diminished the promoting effect of miR-378 on ER stress, as well as its downstream angiogenesis and osteoclastogenesis activities. Tail vein injection of anti-miR-378 lentivirus in an established RA mouse model was shown to ameliorate RA progression. In conclusion, miR-378 amplified RA development by promoting ER stress and downstream angiogenesis and osteoclastogenesis, thus indicating that miR-378 may be a potential therapeutic target for RA treatment.

## Introduction

Rheumatoid arthritis (RA) is a chronic autoimmune disorder marked by pain, inflammation, and discomfort in the synovial joints, predominantly in the hands and wrists. It may also encompass cardiovascular, respiratory, psychological, and bone-related issues [1]. It is believed that a combination of genetic and environmental factors may play a significant role in its development [2, 3]. Individuals suffering from rheumatoid arthritis experience inflammation of the joint capsule, which leads to degeneration of both the underlying bone and cartilage due to the immune system targeting the joints.

Synovial tissue plays a crucial role in maintaining joint health by providing support, nutrients, and lubricants to adjacent cartilage. However, in rheumatoid arthritis (RA), synovial hyperplasia can occur, leading to the abnormal growth of synovial tissue and subsequent deterioration of cartilage and joint surfaces, which can result in joint deformity and dysfunction. In RA, fibroblast-like synoviocytes (FLSs), the main cell population in the synovial tissue, actively participate in the inflammatory process. Inflammatory FLSs undergo uncontrollable proliferation and interact with immune cells like lymphocytes, macrophages, and neutrophils to create an inflammatory microenvironment in the synovium caused by the production of proinflammatory cytokines such as IL-1β, IL-6, and TNF by macrophages. FLSs release vascular endothelial growth factors (VEGF), which can result in the infiltration of more inflammatory cells to the synovitis sites, a chronic inflammatory state. The inflammation condition could further trigger the release of vascular endothelial growth factors (VEGF) by FLSs [4, 5], leading to the formation of new blood vessels, which in turn leads to the infiltration of more inflammatory cells to synovitis sites for maintenance of the chronic inflammatory state. The expression and secretion of receptor activator of nuclear factor Kappa-B ligand (RANKL), and matrix metallopeptidase 13 (Mmp13) were also upregulated in inflammatory FLSs, which caused the subsequent bone erosion and cartilage degeneration, the main characteristic features of RA.

The endoplasmic reticulum (ER) is an organelle mainly offering a finetuned biochemical micro-environment for proper protein folding and membrane transportation [6]. ER stress occurs when there is an accumulation of unfolded or misfolded proteins in the ER, which disrupts its normal function. In response to ER stress, a recovery mechanism called unfolded protein response (UPR) was triggered, which aims to restore ER homeostasis and ensure proper protein folding. In RA, the increased expression of ER stress markers, such as Grp78 and Atf6, has been observed in the synovial tissue of affected individuals, which suggests that ER stress plays a role in the pathogenesis of the disease [7, 8]. As a consequence of ER stress, proinflammatory cytokines released during inflammation in RA can further upregulate the expression of Grp78, which can promote angiogenesis, bone erosion, and synovial cell proliferation, all of which contribute to the progression of RA [4]. Targeting the ER stress response and its regulatory mechanisms has emerged as a promising approach for treating RA.

MicroRNA is short, single-stranded, non-coding RNA sequences that are made up of 21–23 nucleotides and act as a counterpart for targeting and degradation of mRNAs, resulting in the suppression of corresponding protein expression [9]. MicroRNAs are known to play a significant role in the pathogenesis of rheumatoid arthritis (RA). Several miRNAs, including miR-143, miR-145, miR-17-5p, miR-146, and miR-30a, etc., have been found to be dysregulated in RA tissues and are associated with the abnormal phenotype of RA-FLSs (rheumatoid arthritis fibroblast-like synoviocytes) [10]. miR-17-5p has been shown to have anti-inflammatory and anti-erosive effects in the arthritic tissue of RA mice by directly targeting JAK1 and STAT3, two proteins involved in inflammation [11]. Elevated expression of miR-146 has been observed in synovial tissues and suggested as a potential biomarker for RA [12]. However, it’s noted that the complete spectrum of miRNA involvement in the development of RA is still not fully understood. Further research is needed to uncover the roles of other miRNAs and their potential therapeutic implications in RA.

MiR-378, an evolutionarily conserved miRNA, has been demonstrated to be integral in several bone-associated diseases, such as fractures, osteoarthritis, and osteoporosis [13–15]. In our previous study, we have constructed the miR-378 universal transgenic mice (miR-378^high^ mice), and confirmed that miR-378 was involved in OA pathology and associated with inflammation and chondrocyte hypertrophy [13]. In bone homeostasis, miR-378 inhibits BMSCs osteogenesis and bone regeneration by repressing Wnt/β-catenin signaling [14]. On the other hand, clinical studies have noted that a set of miRNAs, including miR-224, miR-760, miR-483-5p, and miR-375, are key players in the development of RA [16]. Interestingly, it was observed that the miR-378 expression level was significantly increased in the synovium of RA patients and was positively correlated with their DAS28 scores, which indicates disease severity. In this study, we intend to uncover the precise role of miR-378 in the progression of RA, potentially shedding light on new therapeutic targets or strategies for the disease.

## Material and methods

### Collagen-induced arthritis (CIA) mouse model

The miR-378 transgenic mice (miR-378^high^ mice) were obtained from Prof. Dahai Zhu’s lab (Chinese Academy of Medical Sciences) and kept in our lab [17]. The genotyping characterization of miR-378^high^ mice was performed as described in our previous study [14]. The C57BL/6 mice were purchased from the Laboratory Animal Service Center (Chinese University of Hong Kong, HKSAR) and included as negative control. For the CIA model, male mice were subcutaneously injected with 0.1 ml of an emulsion containing 100 μg of bovine type II collagen (234184-M, Sigma-Aldrich, St. Louis, MO, USA) and Freund’s complete adjuvant (Chondrex, Washington, USA) into the base of the tail on day 0. After 21 days, a booster injection of 0.1 ml of an emulsion of bovine Collagen II and Freund’s incomplete adjuvant (Chondrex, Washington, USA) was performed as described above. A total of 12 WT and 12 miR-378^high^ mice were randomly divided in half (*n* = 6). Half of the mice were grouped as sham and were injected with PBS, while the other half were named CIA group and were injected with collagen II and Freund’s complete/incomplete adjuvant on day 1 and day 21. The severity of the arthritis was evaluated by measurement of hind paw thickness starting from day 21 to day 35. The clinical arthritis scores were assessed according to the previously mentioned protocol [13]. On day 35, all animals were sacrificed, and samples were collected for further analysis.

### Micro-CT analysis

The mouse hind paws were fixed with 4% paraformaldehyde for 24 h and then kept in 75% ethanol for micro-CT scanning. The high-solution μCT40 (Scanco Medical, Switzerland) with a voltage of 70 kV and a current of 114 μA and 10.5 μm isotropic resolution was applied to scan the microstructure changes of hind paws. The bone reconstruction was reestablished by using the built-in CT software as described previously [18].

### Histological assessment

The hind paws were fixed with 4% paraformaldehyde for 24 h and then decalcification by 10% EDTA buffer (pH 7.4) for 21 days. The paws were then dehydrated, and paraffin-embedded as previously described, and then sectioned into 7 mm thickness for histological analysis. The H&E staining and Safranin O/Fast green staining were applied for evaluating tissue morphology and cartilage degradation as previous protocol described [19].

### TRAP staining

The sections were equilibrated with xylene twice and then sequentially transferred into 100%, 90%, 70% ethanol, and H_2_O, respectively, for deparaffinization. The commercially available TRAP staining kit (Sigma-Aldrich, MO, USA) was applied for the sample staining according to the manufacturer’s instructions. Trap-positive multinucleated cells were counted as mature osteoclasts, and photos were taken by Leica DMIRB Inverted Leica Modulation Contrast Microscope (Leica, Wetzlar, Germany).

### Immunofluorescence and immunohistochemistry

The immunofluorescence (IF) and immunohistochemistry (IHC) staining were performed according to the standard protocols. Basically, the sections were boiled in 100 °C citrate buffer for antigen retrieval and then soaked in hydrogen peroxide for quenching. After that, the sections were blocked with 3% BSA and incubated with primary antibody including rabbit anti-IL-1 (1:100, YT2322, Immnoway, USA), rabbit anti-Grp78 (1:100, ab21685, Abcam, USA), rabbit anti-Atf6 (1:100, ab37149, Abcam, USA), rabbit anti-VEGF (1:100, ab46154, Abcam, USA) and rabbit anti-CD31 (1:100, Ab182981, Abcam, USA) at 4 °C overnight. The sections were washed with PBS and conjugated with a secondary antibody, goat Anti-Rabbit IgG H&L (ab97047, Abcam, Cambridge, UK). The horseradish peroxidase-streptavidin system (Dako, USA) was applied for signal visualization, which was followed by counterstaining with hematoxylin. For the IF staining, the secondary antibodies were replaced by goat anti-rabbit IgG-H&L (Alexa Fluor® 488) (ab150077, Abcam, Cambridge, UK), or goat anti-rabbit IgG-H&L (Alexa Fluor® 647) (ab150083, Abcam, Cambridge, UK). The photographs of selected areas were recorded under a Leica DMIRB Inverted Leica Modulation Contrast Microscope (Leica, Wetzlar, Germany).

### Isolation and primary culture of FLSs and siRNA knockdown

The mice were sacrificed using cervical dislocation, the ankle joint was opened under aseptic conditions, the synovial layer was separated and isolated using surgical scissors. The synovium tissue was washed three times with 4°C PBS containing 1% PSN (Life Technology, New York, USA), and then minced and digested with 0.25% trypsin (Thermo Fisher Scientific, Mass, USA) and 1% type II collagenase (17101015, Thermo Fisher Scientific, Mass, USA) respectively. After that, the cells were collected and cultured in Dulbecco’s modified Eagle’s medium (DMEM) containing 10% fetal bovine serum and 1% antibiotics in 5% CO_2_ at 37 °C for 3 passages, the IF staining was used to identify the isolated FLSs (Supplementary Fig. 1). For siRNA knockdown, The Crebrf small interfering RNAs (si-Crebrf) and control siRNAs (si-NC) were synthesized by GenePharma (Shanghai, China). The transient transfection was performed using Lipofectamine 3000 (Invitrogen, USA) following the previous study [20].

### Endoplasmic reticulum (ER) stress stimulation of synoviocytes

Morphological analysis of FLSs under ER stress induction was performed with transmission electron microscopy (TEM). Basically, the WT FLSs and miR-378^high^ FLSs were cultured and stimulated with TNFα (10 ng/ml) or 5 μM thapsigargin (Tg) for 12 h. The cells were then fixed with 2.5% glutaraldehyde in 0.1 M cacodylate buffer at 4 °C. The cells were pelleted and further fixed within 2% osmium tetroxide (Sigma-Aldrich, MO, USA) dissolved in 0.1 M cacodylate buffer for 2 h at room temperature, and then washed with distilled water. After dehydration, a series of 1:2, 1:1, and 1:2 mixtures of acetone–Epon resin (EMBed Resin 812, EMS) and 100% resin were applied for infiltration. Subsequently, cell pellets were then saturated with the same resin in BEEM capsules (Ted Pella, Ca, USA) and polymerized at 55 °C for 48 h. The block sections at 60-nm thickness were obtained and stained with lead citrate (Merck KGaA, Darmstadt, Germany) for 5 min and observed under a transmission electron microscope at a beam voltage of 100 kV (H7700, Hitachi, Chiyoda-ku, Tokyo, Japan).

### Tube formation assay

Endothelial tube formation test was performed for evaluating angiogenic activity in vitro according to our described previously [21]. In brief, the Matrigel Matrix (Corning, NY, USA) was coated at the bottom of 96-well plates at 37 °C for 1 h for gelling promotion. Then, the Human umbilical vein endothelial cells (HUVEC) were seeded at the concentration of 4 × 10^4^ cells/well. Subsequently, the culture medium was replaced with a conditioned medium obtained from WT and miR-378^high^ FLSs culture. After 3- and 6-h incubation, the plate was observed and photographed under an inverted phase-contrast microscopy (Nikon, Tokyo, Japan). The tube-like structures in each well were evaluated by measuring the branching length and number of conjunctions.

### Osteoclastogenesis

Raw 264.7 cells were seeded in a 24-well plate at the density of 5 × 10^4^ cells/cm^2^. A conditioned medium of RA FLSs from WT or miR-378^high^ mice was applied to the cells for osteoclastogenesis induction. After 5-day induction, the cells were fixed in 4% paraformaldehyde for 10 min and stained with a Tartrate-resistant acid phosphatase (TRAP) activity kit according to the manufacturer’s instructions (386A, Sigma-Aldrich, USA). The TRAP-positive multinucleated (nuclei > 3) cells were scored as osteoclasts under a light microscope.

### Luciferase reporter assay

Mouse miR-378-3p mimics were purchased from GenePharma Company (Shanghai, China). pmiRGLO-Crebrf wide type (wt) and site-mutation (mut) recombinant vectors were constructed by Genscript Biotech Company (Nanjing, China). Renilla luciferase control reporter vectors were purchased from Upstate Cell Signaling (CST, NY, USA). Dual-luciferase assay was performed following the previous protocol [14]. HEK293 cells were transfected with pmiRGLO-Crebrf wt or mut vectors together with miR-378-3p mimics. The renilla luciferase vector was co-transfected as a normalization control. The luciferase activities were measured using PerkinElmer VictorTM X2 2030 multilabel reader (Waltham, USA). The renilla luciferase activity was also measured for normalization.

### Real-time PCR

For RNA extraction of cells or synovium, the TRIzol Reagent (Invitrogen) was applied following the manufacturer’s protocol. The PrimeScript RT Master Mix reverse transcription kit (TaKaRa, Japan) was applied to reverse transcript the RNA into cDNA. The polymerase chain reaction was processed by using SYBR Green PCR Master Mix (Applied Biosystems, Foster, CA, USA) on the StepOnePlus RT-PCR system (Applied Biosystems). The real-time PCR primers were synthesized by Life Technologies (CA, USA) and listed in Supplementary Table 1. The gene expression level was evaluated using 2^−ΔΔCT^ method following the previous protocol and expressed as fold change relative to the controls.

### Western blot

For tissue protein collection, the synovial tissues were carefully dissected and were homogenized with a rotary homogenizer in radioimmunoprecipitation assay (RIPA) buffer supplemented with complete Mini Protease Inhibitor Cocktail (Roche, Basel, Swiss). Total protein from cultured cells was in situ lysed by radioimmunoprecipitation assay (RIPA) buffer supplemented with complete Mini Protease Inhibitor Cocktail (Roche, Basel, Swiss). The protein fraction, electrophoresis, and electroblotting were performed as described before [22]. The primary antibodies used include rabbit anti-RANKL (1:3000, ab9957, Abcam, USA), rabbit anti-Crebrf (1:250, sc-393012, Santa Cruz, USA), rabbit anti-β-actin (1:3000, Sc-47778, Santa Cruz, USA) and other Grp78, Atf6, VEGF, and antibodies as described in IHC&IF sections with 1:3,000 dilution. The horseradish-peroxidase (HRP)-conjugated secondary antibody (1:5000, CST7074, Cell signaling technology, Danvers, MA, USA) was used to incubate the membrane for an additional 2 h. Finally, the signals of the target protein were visualized by an enhanced chemiluminescence reagent (Thermo Fisher Scientific, IL, USA).

### Statistical analysis

A priori power analysis was performed to calculate the sample size using G*Power 3.0.10 (www.gpower.hhu.de). The sample size was determined by using the criteria of 0.05 statistical level of significance, size effect of at least 1, and statistical power of 0.80. All data were presented as mean ± standard deviation. Statistical significance comparing two independent groups with parametric data was assessed by unpaired Student t-test. All data analysis and staining were performed under double-blind conditions. The analysis was performed with GraphPad Prism 8 (GraphPad Software, USA). We considered evidence of significant effects at *p* values < 0.05.

## Results

### MiR-378^high^ mice displayed exaggerated RA phenotype in collagen-induced arthritis (CIA) model

**Fig. 1:**
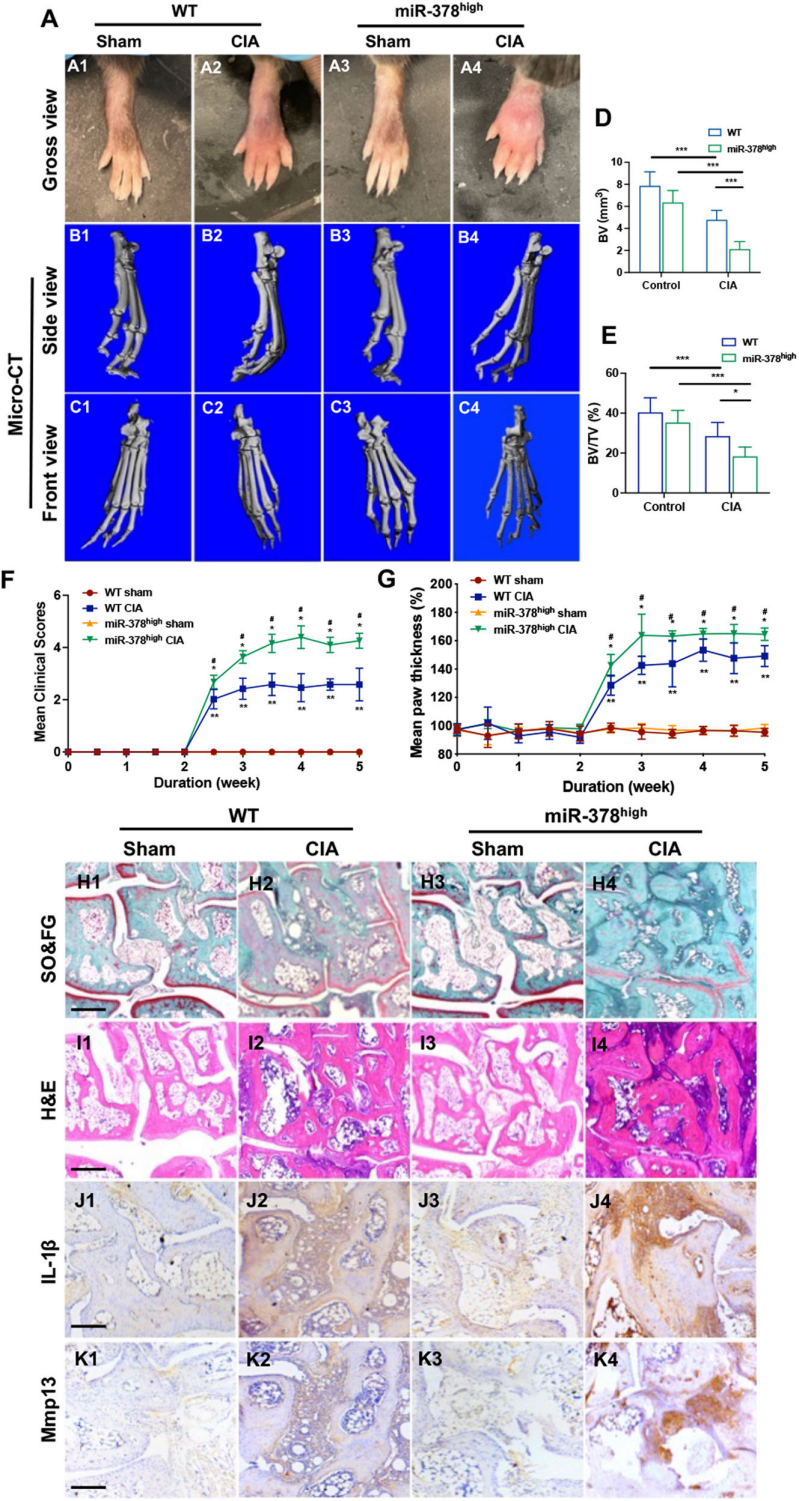
MiR-378^high^ CIA mice displayed exaggerated RA phenotype of synovium in collagen-induced arthritis (CIA) model. **A** Representative paw photographs of WT and miR-378^high^ mice after PBS or Collagen II induction. **B**, **C** The side view (**B**) and front view (**C**) of micro-CT reconstruction of mice hind paw. **D**, **E** Semiquantitative analysis of bone volume (BV) (**D**) and bone volume/total volume (BV/TV) (**E**) of mice hind paw bone from different groups (*n* = 6; *p < 0.05, **p < 0.01, ***p < 0.001). **F**, **G** Mean clinical scores (**F**) and mean paw thickness (**G**) from different groups were assessed every day after collagen stimulation (*n* = 6; *p < 0.05, **p < 0.01, CIA versus sham; ^#^*p* < 0.05, ^##^*p* < 0.01, miR-378^high^ group versus WT group). **H**, **I** Ankle histopathology analysis by Safranin O&fast green (**H**) and H&E staining (**I**). Scale bars: 100 μm. **J**, **K** Immunohistochemistry analysis of RA ankle using anti-IL-1β (**J**) and anti-Mmp13 (**K**) antibodies. Scale bars: 100 μm.

Both WT and miR-378^high^ mice were subjected to a 30-day induction of type II collagen arthritis. After 4 weeks, the hind paws were collected and used for micro-CT analysis. The CIA animals in both the miR-378^high^ group and WT group exhibited joint swelling and bone structural deformities. However, the miR-378^high^ group showed more severe symptoms compared to the WT group, suggesting that miR-378 may exacerbate RA (Fig. 1A–C). Additionally, a semi-quantitative analysis of bone volume (BV) and bone volume fraction (BV/TV) during CIA induction confirmed the detrimental impact of miR-378 overexpression on arthritis development (Fig. 1D, E). Consequently, miR-378 overexpression accelerated RA development, as quantitatively demonstrated by the average clinical score and average paw thickness measurements. The pathological symptoms of RA appeared two weeks after collagen injection and plateaued by week 3, as indicated by the mean clinical score and mean paw thickness. However, miR-378 overexpression expedited the progression of RA, resulting in a doubling of the average clinical score and a 1.2-fold increase in average paw thickness (Fig. 1F, G). Safranin O&fast green and H&E staining revealed that collagen treatment significantly caused cartilage degradation and damage to the extracellular matrix, leading to inflammatory hyperplasia between the joints, as observed by H&E staining (Fig. 1H, I). Furthermore, the expression level of IL-1β and Mmp13 in the synovial membrane of the miR-378^high^ group was higher upon collagen stimulation, triggering inflammation in the FLSs and accelerating joint deterioration and bone resorption (Fig. 1J, K, Supplementary Fig. 2).

### MiR-378^high^ CIA mice showed more severe ER stress as well as angiogenesis and osteoclastogenesis

**Fig. 2:**
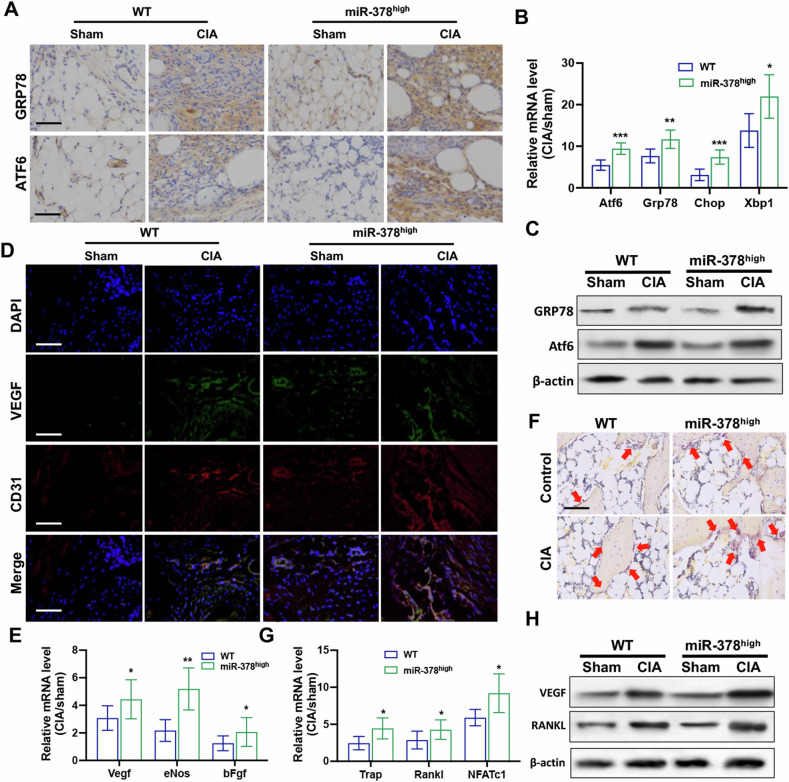
miR-378 overexpression severely induced ER stress in FLSs as well as the consequent angiogenesis and osteoclastogenesis. **A** Representative immunohistochemical staining of ER stress-related markers in the synovium sections obtained from miR-378^high^ CIA mice. Scale bars: 100 μm. **B**, **C** Relative mRNA (**B**) and protein (**C**) expression level of ER stress-related markers in synovium sections from miR-378^high^ CIA mice (*n* = 6; *p < 0.05, **p < 0.01, ***p < 0.01). **D** Representative immunofluorescence staining of angiogenesis-related marker VEGF and CD31 in the synovium sections obtained from miR-378^high^ CIA mice. Scale bars: 100 μm. **E** Relative mRNA expression level of angiogenesis markers in synovium tissues from miR-378^high^ CIA mice (*n* = 6; *p < 0.05, **p < 0.01). **F** Trap staining of calcaneus sections obtained from miR-378^high^ CIA mice. Scale bars: 100 μm. **G** Relative mRNA expression level of osteoclastogenesis markers in synovium tissues from miR-378^high^ CIA mice (*n* = 6; *p < 0.05). **H** Protein expression pattern of VEGF and RANKL in synovium tissues from miR-378^high^ CIA mice.

ER stress is crucial for synoviocyte proliferation, angiogenesis, and bone erosion, which are the pathological hallmarks of RA. In this study, ER stress levels were assessed under control and CIA conditions in joint tissues of both the WT and miR-378^high^ mice. Upon collagen induction, the protein expression levels of ER stress markers, including Grp78 and Atf6 were significantly increased. This increase was further amplified by miR-378 overexpression, as demonstrated by IHC staining (Fig. 2A). Quantitative real-time PCR and Western blot analysis also indicated the same expression pattern of ER stress markers among different groups (Fig. 2B, C). Furthermore, synovial tissue was collected for angiogenetic activity analysis. The IF staining results indicated that the expression levels of the angiogenic marker VEGF and the vascular endothelial marker CD31 were both increased in synovial tissues after collagen treatment. This increase was further enhanced under miR-378 overexpression (Fig. 2D). The conclusion was further strengthened by real-time PCR and Western blot analysis of VEGF and CD31 expression levels (Fig. 2E, H). The Trap staining also indicated exaggerated bone resorption in the calcaneus of the miR-378^high^ group compared with the WT group under collagen induction conditions (Fig. 2F). This was also confirmed by mRNA and protein expression assays of osteoclastogenic markers (Fig. 2G, H).

### MiR-378 intensified proinflammatory factor and thapsigargin-induced ER stress in fibroblast-like synoviocytes

**Fig. 3:**
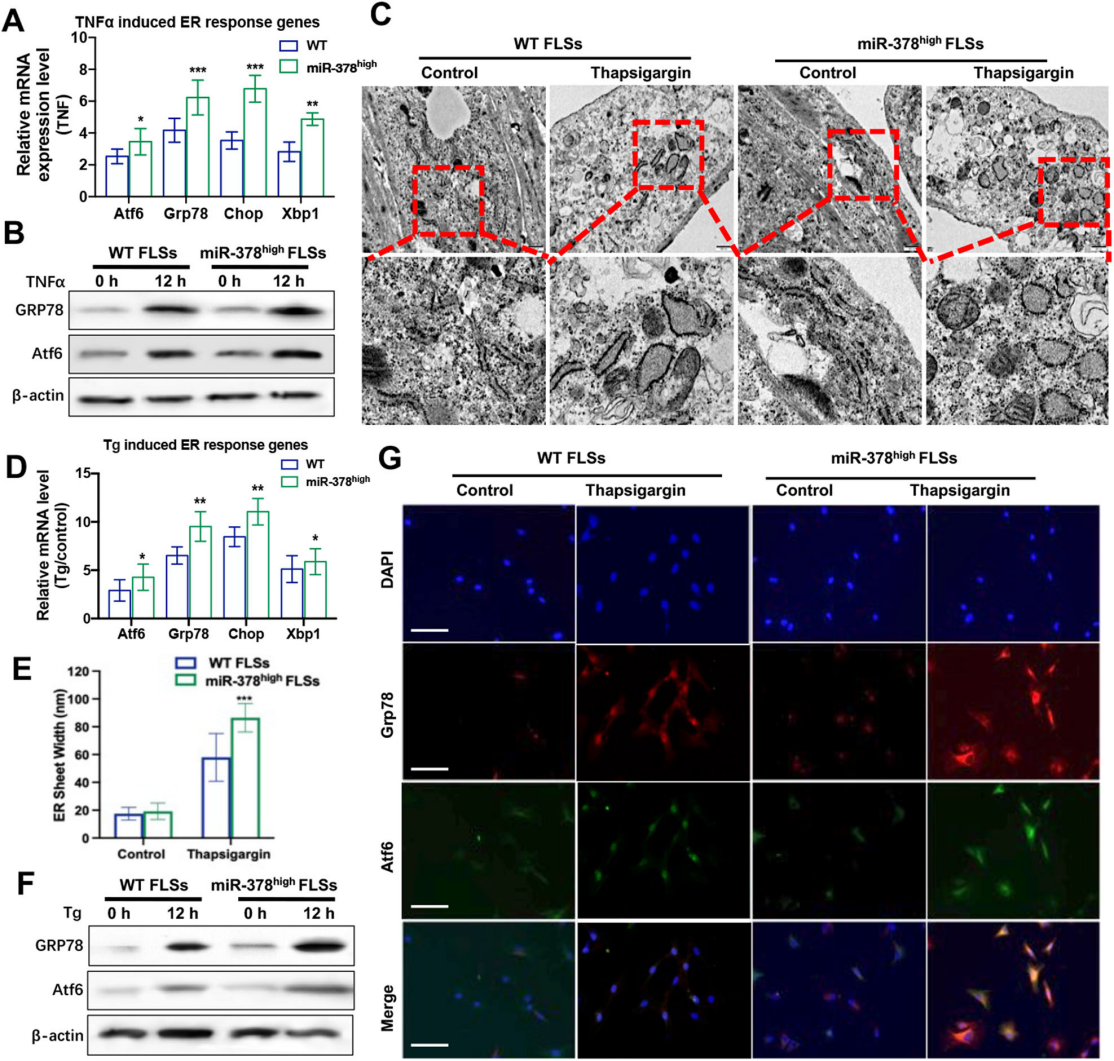
Induction of ER stress-related makers by proinflammatory cytokines was aggregated upon miR-378 overexpression. **A**, **B** The mRNA (**A**) and protein (**B**) expression level of ER stress-related makers in RA-FLSs from WT and miR-378^high^ mice upon TNFα activation (*n* = 6; *p < 0.05, **p < 0.01, ***p < 0.001). **C** Both WT and miR-378^high^ FLSs showed ER stress after TG induction, as manifested by enlarged endoplasmic reticulum cisternae. **D** Semi-quantitative analysis of ER sheet width. **E**, **F** The mRNA (**E**) and protein (**F**) expression level of ER stress-related makers in RA-FLSs from WT and miR-378^high^ mice upon thapsigargin stimulation (*n* = 6; *p < 0.05, **p < 0.01). **G** Immunofluorescence staining of ER stress-related markers in RA-FLSs from WT and miR-378^high^ mice upon thapsigargin stimulation. Scale bar: 100 μm.

The ER stress response could be stimulated by the high expression of the pro-inflammatory element TNFα in the microenvironment of rheumatoid arthritis (RA). The ER stress response in fibroblast-like synoviocytes (FLSs) was significantly elevated by TNFα treatment. Additionally, FLSs isolated from miR-378^high^ mice displayed a higher ER stress response compared with WT mice under TNFα stimulation, as demonstrated by the transcriptional and translational levels of ER stress response markers (Fig. 3A, B). Thapsigargin (Tg), a suppressor of ER calcium ATPase, could hinder calcium transport into the ER, subsequently resulting in ER stress and the unfolded protein response (UPR). Compared with the WT group, FLSs from miR-378^high^ mice demonstrated enlarged endoplasmic reticulum cisternae (Fig. 3C, D). Real-time PCR, Western blot analysis, and IF staining results also demonstrated that expression of ER stress markers was all increased by Tg stimulation and further exacerbated upon miR-378 overexpression (Fig. 3E–G).

### Angiogenesis and osteoclastogenesis activities were increased by RA miR-378^high^ FLSs conditioned medium

**Fig. 4:**
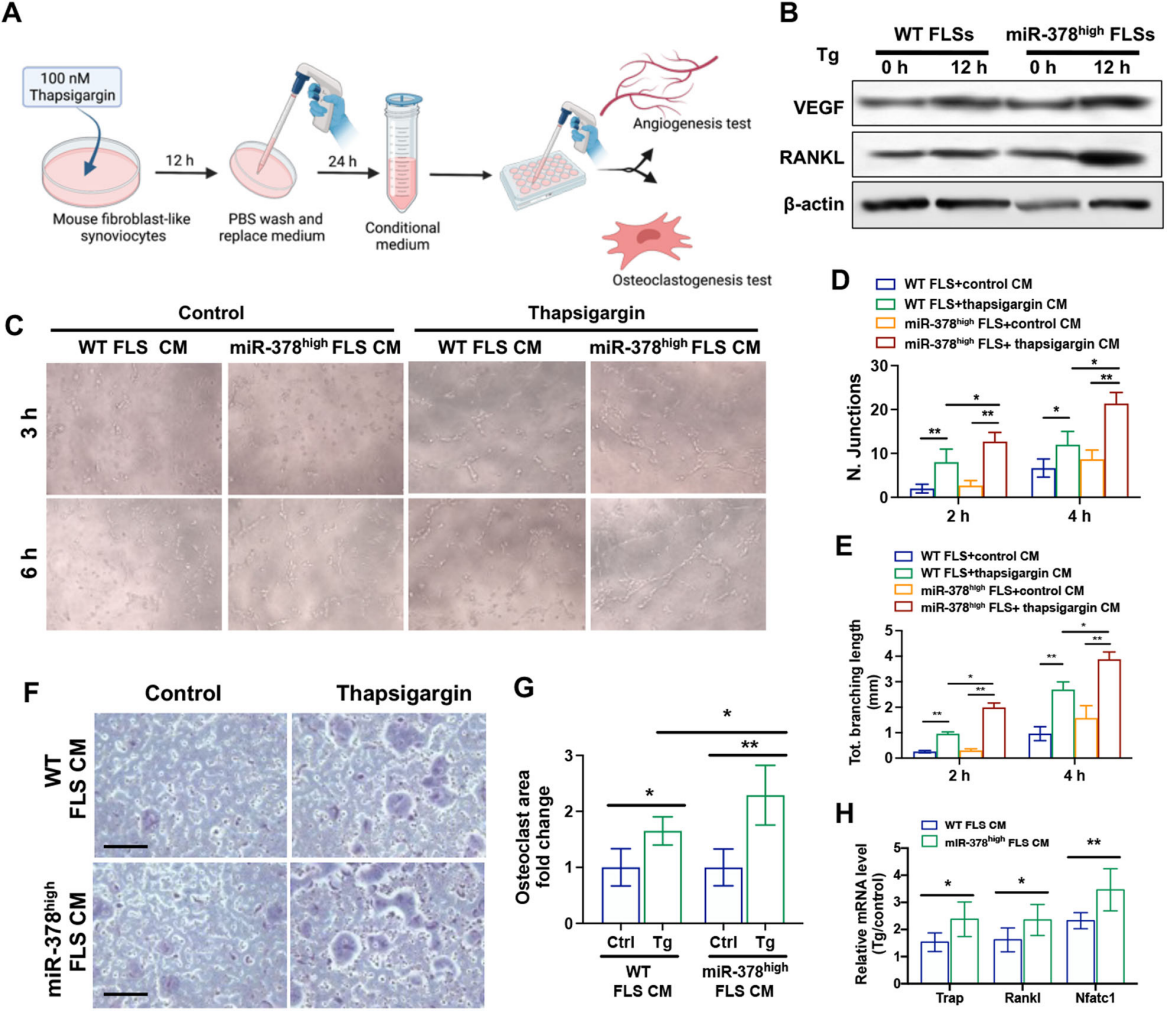
ER stressor-induced miR-378^high^ FLSs promoted angiogenesis and osteoclastogenesis. **A** Diagram to show the collection of conditioned medium from RA-FLSs and the co-culture system for angiogenesis and osteoclastogenesis induction. **B** The protein expression level of angiogenesis and osteoclastogenesis makers in RA-FLSs from WT and miR-378^high^ mice upon thapsigargin activation. **C** Images of tube formation at 3 and 6 h post-induction with the treatment of conditioned medium from miR-378^high^ FLSs upon thapsigargin stimulation. **D**, **E** Semiquantitative analysis of total branching length of each group (**D**) and number of junctions (**E**) in each group (*n* = 6; *p < 0.05, **p < 0.01). **F** TRAP staining assay at day 5 with the treatment of conditioned medium from miR-378^high^ FLSs upon thapsigargin stimulation. Scale bar: 100 μm. **G** Quantitative data of osteoclast fold change (*n* = 6; *p < 0.05, **p < 0.01). **H** The mRNA expression level of osteoclastogenesis-related markers measured by qRT-PCR (*n* = 6; *p < 0.05, **p < 0.01).

To investigate the promoting activity of miR-378^high^ FLSs on angiogenesis and osteoclastogenesis, mouse FLSs were induced with 100 nM of Tg to activate the ER stress response. Subsequently, the conditioned medium (CM) of miR-378^high^ FLSs was collected and co-cultured with human umbilical vein endothelial cells (HUVEC) and macrophage cells Raw 264.7, respectively (Fig. 4A). Under Tg induction, miR-378^high^ FLSs exhibited higher protein expression levels of VEGF and RANKL compared to WT FLSs (Fig. 4B). The CM of miR-378^high^ FLSs stimulated with Tg demonstrated a stronger promoting effect on tube formation ability of HUVECs compared to WT FLSs (Fig. 4C). The quantitative analysis of total branching length and number of junctions further supported the effect of miR-378^high^ FLSs on angiogenesis activity. The angiogenic capacity of HUVEC cells treated with the CM obtained from miR-378^high^ FLSs was approximately 1.3–2.0 times greater than the WT FLSs CM group (Fig. 4D, E). On the other hand, a significant increase in osteoclast formation of RAW264.7 cells was observed when treated with Tg-induced FLSs CM, and this increase was further amplified by miR-378 overexpression. The CM obtained from miR-378^high^ FLSs under Tg stimulation exhibited a greater osteoclastogenic promoting capability, approximately 1.5 times greater compared to WT FLSs with the Tg treatment group (Fig. 4F, G). Additionally, the transcriptional levels of osteoclastogenic markers, including Trap, c-Fos, and Nfatc1, were also enhanced in the CM of FLSs after Tg treatment and further increased by miR-378 overexpression (Fig. 4H).

### MiR-378 regulated ER stress by directly targeting Crebrf in RA-FLSs

**Fig. 5:**
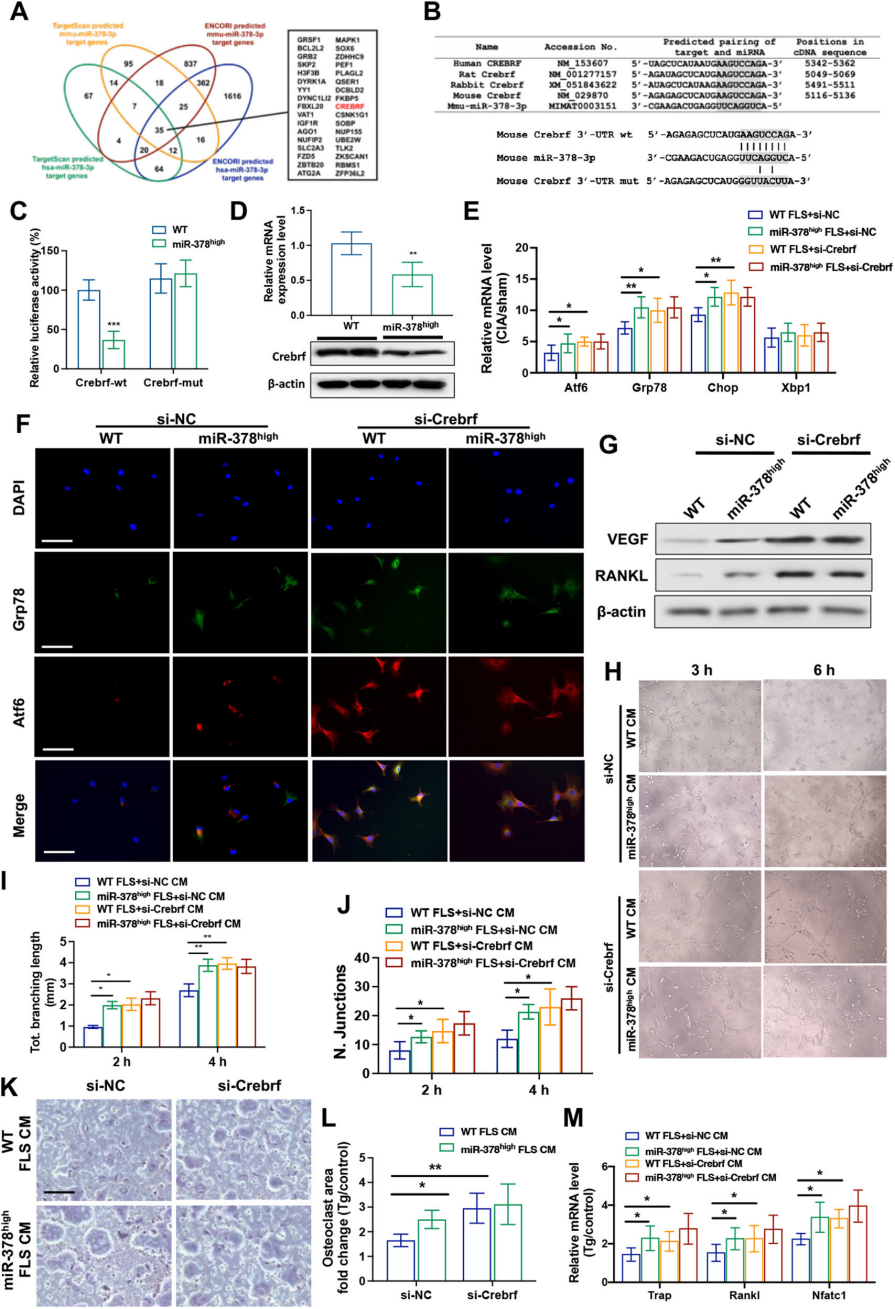
Crebrf was characterized to be the key hub gene in miR-378 regulated ER stress in FLSs and mediated the promoting effect of miR-378^high^ FLSs on angiogenesis and osteoclastogenesis. **A** Schematic diagram to show the miRNA target gene screening procedure. The TargetScan and ENCORI software were applied for in silico analysis of conserved 3’-UTR binding sites of miR-378 target genes, respectively. The overlapped genes were listed. **B** Conservation of the miR-378-3p binding site on Crebrf 3′-UTR (shaded region) among different species. The wild type (wt) and mutation (mut) form of the Crebrf 3′-UTR luciferase reporter vector were shown. **C** Effects of miR-378-3p on the luciferase activity of pmiRGLO vectors incorporated with Crebrf-wt or Crebrf-mut sequence were measured (*n* = 6; ***p < 0.001). **D** The mRNA and protein expression levels of Crebrf in WT and miR-378 synoviocytes were measured respectively (*n* = 6; *p < 0.05). **E** The mRNA expression levels of ER stress markers of WT and miR-378^high^ synoviocytes upon Crebrf knockdown under CIA condition were assessed by real-time PCR (*n* = 6; *p < 0.05, **p < 0.01, ***p < 0.001). **F** Representative immunofluorescence staining of ER stress markers Grp78 and Atf6 upon Crebrf knockdown in FLSs obtained from miR-378^high^ CIA mice. Scale bar: 100 μm. **G** Western blot analysis of VEGF and RANCL protein expression in miR-378^high^ FLSs upon Crebrf knockdown. **H** Images of tube formation at 3 and 6 h post-induction with the treatment of conditioned medium from miR-378^high^ FLSs upon Crebrf knockdown. **I**, **J** Semiquantitative analysis of total branching length of each group (**I**) and number of junctions (**J**) in each group. **K** TRAP staining assay at day 5 with the treatment of conditioned medium from miR-378^high^ FLSs upon Crebrf knockdown. Scale bar: 100 μm. **L** Quantitative data of osteoclast fold change (*n* = 6; *p < 0.05, **p < 0.01). **M** The mRNA expression level of osteoclastogenesis-related markers measured by qRT-PCR (*n* = 6; *p < 0.05, **p < 0.01, ***p < 0.001).

To study the underlying mechanism of miR-378 in regulating RA pathogenesis, two analysis tools, Targetscan and ENCORI, were utilized to predict the direct downstream targets of miR-378-3p. Human and mouse miR-378 were included to characterize the target proteins with conserved binding sequences between the two species. Among the common targets of the two prediction software, Crebrf was selected as the putative direct target of miR-378 due to its high involvement in ER stress response (Fig. 5A). The conservation of miR-378-3p binding sites on the 3′-UTR of Crebrf was further confirmed among different species, including human, rabbit, rat, and mouse. The wild-type (WT) and site-directed mutation constructs of the Crebrf 3′-UTR luciferase reporter vector were also demonstrated (Fig. 5B). The overexpression of miR-378 significantly reduced the luciferase activity of the Crebrf wt reporter system, while this reduction was abolished in the Crebrf mut reporter system (Fig. 5C). The mRNA and protein expression levels of Crebrf were also reduced in miR-378^high^ FLSs compared with WT FLSs (Fig. 5D). The expression levels of ER stress markers were increased in miR-378^high^ FLSs compared with the WT group, but this increase was diminished with siRNA-induced Crebrf knockdown (Fig. 5E). The result of the IF study further proved that the intensifying effectiveness of miR-378 on ER stress was substantially abolished when Crebrf was silenced, as evidenced by the protein expression levels of Grp78 and Atf6 (Fig. 5F).

### Crebrf mediated the promoting effect of RA-FLSs from miR-378^high^ mice on angiogenesis and osteoclastogenesis

The regulating effect of Crebrf on miR-378^high^ FLS-promoted angiogenic and osteoclastogenic activities was also examined. The expression of Crebrf in both WT and miR-378^high^ FLSs was diminished by siRNA knockdown. Afterward, the FLSs were induced with Tg, and the CM was collected for angiogenesis and osteoclastogenesis assays. Crebrf knockdown resulted in a significant increase in the protein levels of VEGF and RANKL in FLSs (Fig. 5G). HUVECs treated with CM from miR-378^high^ FLSs showed improved tube formation, number of junctions, and total branch length compared with the WT FLSs group. However, the amplifying impact of miR-378 overexpression was reduced upon Crebrf silencing, suggesting that Crebrf-regulated ER stress response was involved in the angiogenesis-promoting activity of miR-378^high^ FLSs (Fig. 5H–J). On the other hand, CM from miR-378^high^ FLSs significantly increased the osteoclastogenesis of Raw 264.7 cells compared with WT FLSs. Upon Crebrf knockdown, the CM collected from FLSs of both groups showed similar high osteoclastogenic promoting activities to Raw 264.7 cells, as indicated by the quantitative analysis of osteoclast area fold change and the measurement of mRNA expression level of osteoclast formation markers (Fig. 5K–M).

### Anti-miR-378 injection ameliorated RA phenotype in synovium in miR-378^high^ CIA mice

**Fig. 6:**
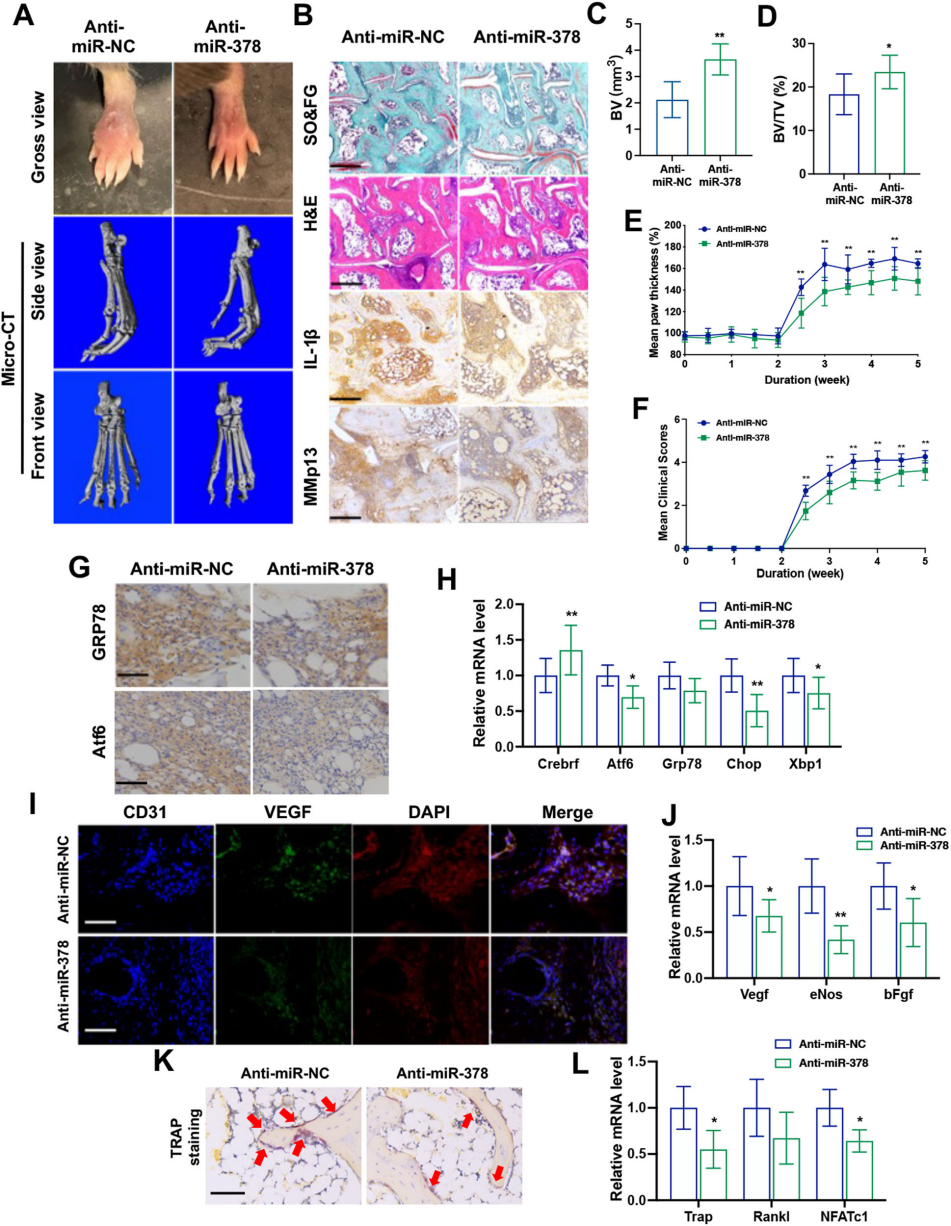
Anti-miR-378 injection ameliorated RA phenotype in synovium in miR-378^high^ CIA mice. **A** Representative paw photographs and the side view and front view of micro-CT reconstruction of hind paw of miR-378^high^ mice after anti-miR-NC or anti-miR-378 mediated lentivirus treatment. **B** Histopathology analysis of RA joints by Safranin O&fast green and H&E staining, as well as immunohistochemistry staining using anti-IL-1β and anti-Mmp13 antibodies. Scale bars: 100 μm. **C**, **D** Semiquantitative analysis of bone volume (BV) (**C**) and bone volume/total volume (BV/TV) (**D**) of mice hind paw bone from different groups (*n* = 6; *p < 0.05, **p < 0.01). **E**, **F** Mean clinical scores (**E**) and mean paw thickness (**F**) from different groups were assessed every day after collagen stimulation (*n* = 6; **p < 0.01). **G** Representative immunohistochemical staining of ER stress-related markers in the synovium sections after anti-miR-378 lentivirus treatment. Scale bars: 100 μm. **H** Relative mRNA expression level of ER stress-related markers in synovium sections (*n* = 6; *p < 0.05, **p < 0.01). **I** Representative immunofluorescence staining of angiogenesis marker VEGF and CD31 in the synovium sections. Scale bars: 100 μm. **J** Relative mRNA expression level of angiogenesis markers in synovium tissues (*n* = 6; *p < 0.05, **p < 0.01). **K** Trap staining of synovium sections. Scale bars: 100 μm. **L** Relative mRNA expression level of angiogenesis markers in synovium tissues (*n* = 6; *p < 0.05).

Lentivirus harboring the anti-miR-378 sequence was tail-vein injected into the miR-378^high^ CIA mouse model. Upon treatment, both gross view and micro-CT images showed amelioration in joint swelling and deformity (Fig. 6A). Comparing with the anti-miR-NC control group, anti-miR-378 treatment significantly rescued joint cartilage erosion and maintained the extracellular matrix, while repressing the expression of the pro-inflammatory factor, IL-1β, and the ECM degradation enzyme, Mmp13 (Fig. 6B). Anti-miR-378 treatment also elevated BV and BV/TV of mice hind paws during arthritis development (Fig. 1C, D). Furthermore, the assessment of swollen joint count and arthritis index also solidified the amelioration of anti-miR-378 treatment quantitatively (Fig. 6E, F). Transcription and translation levels of ER stress markers in the synovium of miR-378^high^ CIA mice were significantly downregulated by anti-miR-378 treatment (Fig. 6G, H). Meanwhile, anti-miR-378 exerted a therapeutic role in anti-angiogenesis and anti-osteoclastogenesis, as demonstrated by a significant decrease in the protein and mRNA expression of angiogenic markers, VEGF and CD31, as well as osteoclastogenic markers, including RANKL and Trap (Fig. 6I–L).

## Discussion

In this study, we investigated the role of miR-378 in the development and progression of rheumatoid arthritis (RA) using a collagen-induced arthritis (CIA) mouse model. We found that miR-378 overexpression leads to accelerated joint swelling and deformation upon collagen induction. Furthermore, we observed that miR-378^high^ mice exhibited increased extracellular matrix degradation and microstructure collapse upon collagen treatment, leading to the accelerated progression of RA pathology in the joint cavity. In terms of mechanism, we observed elevated expression of ER stress markers, specifically Grp78 and Atf6, as well as angiogenic markers VEGF and CD31, and osteoclastogenic markers RANKL and Trap, particularly in miR-378^high^ CIA mice. In vitro studies using fibroblast-like synoviocytes (FLSs) showed that miR-378^high^ FLSs had higher levels of ER stress when induced with TNFα or thapsigarin, as compared to WT FLSs. We also found that the expression level of mitochondrial oxidative phosphorylation (OxPhos)-related genes were repressed, while autophagy-related genes were increased in miR-378^high^ FLSs, which further suggested the increased ER stress may induce mitochondrial respiratory capacity impairment and mitochondrial autophagy (Supplementary Fig. 3). The CM from miR-378^high^ FLSs induced higher angiogenic and osteoclastogenic activities compared to that from WT FLSs. Further investigation revealed that miR-378 inhibited the expression of Crebrf, which is involved in ER stress regulation. Inhibition of Crebrf led to the release of its binding protein Creb3, a stimulator of ER stress. Silencing of Crebrf diminished the promoting effects of miR-378 on ER stress in FLSs, as well as the downstream angiogenic and osteoclastogenic activities. To validate the potential therapeutic implications, we injected lentiviral-mediated miR-378 antisense into the miR-378 overexpressing RA model, which resulted in a significant rescue of the progression of RA. Overall, this study provides insights into the role of miR-378 in RA pathogenesis, promoting ER stress, angiogenesis, and osteoclastogenesis.

During ER stress, the glucose-regulated protein of 78 kDa (GRP78), a molecular chaperone also known as binding immunoglobulin protein (BiP), initiates a signaling cascade of UPR (unfolded protein response). Following initiation by GRP78, the main UPR signaling cascade is mediated by three ER-localized transmembrane sensors: inositol-requiring transmembrane kinase-endoribonuclease-1α (IRE1α), double-stranded RNA-dependent protein kinase-like ER kinase (PERK), and activating transcription factor 6 (ATF6) [23]. The ER stress response is implicated in RA pathologies by regulating the production of pro-inflammatory cytokines. Rheumatoid FLSs and macrophages derived from the synovial fluid of RA patients exhibited significant activation of the IRE1/XBP1 axis, which is a prerequisite for the production of inflammatory cytokines [23, 24]. ATF6 was also found to be significantly increased in the RA synovium, contributing to elevated viability and cytokine production in RA-FLSs [25]. All these studies highlighted the significant role of ER stress in RA pathogenesis. Our study also revealed that miR-378 exacerbates ER stress, leading to increased RA severity and cytokine production. This discovery points to a novel therapeutic pathway for RA.

Upon ER stress activation, the classical UPR signaling pathway could generate angiomodulatory or angiostatic signals. All three branches of UPR, including IRE1α, PERK, and ATF6 could increase the expression of the key angiogenesis marker VEGF. Moreover, increased VEGF has been shown to reciprocally promote the expression of IRE-1 and Grp78/Bip, along with reactive oxidant species (ROS) production in HUVECs [26]. ER stress-initiated angiogenesis is highly activated in RA. ER stress can trigger the formation of novel blood vessels, thereby increasing nutrient and oxygen supply, as well as the delivery of inflammatory cells and cytokines to synovial tissues [27, 28]. Moreover, angiogenesis plays a crucial role in the proliferation of synovial cells in RA, leading to synovial hyperplasia and the formation of pannus tissue. These processes further contribute to cartilage erosion and bone destruction [29].

On the other hand, ER stress was also highly related to bone homeostasis via UPR signaling pathways [24, 30]. The disruption of bone homeostasis leads to osteoclastogenesis in RA joints. Two branches of UPR, IRE1, and PERK were involved in osteoclast differentiation. It has been observed that RANKL significantly activates the IRE1/XBP1 signaling pathway during the early stages of osteoclast differentiation [31]. Guo et al. also reported that PERK inhibition could downregulate osteoclast formation and bone resorption [32]. Furthermore, previous research suggests that UPR signaling branches, including PERK, ATF6, and IRE1/XBP1, can all promote RANKL expression in osteoblasts, indirectly enhancing osteoclastogenesis [33].

In our study, we found that ER stress induced by proinflammatory cytokines or the ER stress inducer thapsigargin was elevated upon miR-378 overexpression. This, in turn, further stimulated downstream angiogenesis and osteoclast differentiation, respectively. MiRNAs are widely involved in the pathogenesis of RA through various regulatory mechanisms. MiR-150-5p has been proposed as a regulator of immune diseases and has been associated with the onset and progression of various immune diseases, including RA. MiR-150-5p was reported to directly target the expression of SOCS1, a key RA marker in FLSs, and repress its proliferation [34]. Another lncRNA sponge study also revealed THBS2 as the direct target of miR-150-5p, which further regulates ER stress-related TLR4/NF-κB pathway in RA-FLSs apoptosis [35]. Furthermore, MSC-derived miRNA-150-5p-enriched exosomes have shown a therapeutic effect on RA by directly modulating MMP14 and VEGF expression, consequently promoting angiogenesis [36]. Additionally, miR-708-5p has been implicated in RA pathogenesis by promoting apoptosis in FLSs through a Wnt3a/β-catenin-dependent mechanism [37]. Specifically, miR-34a-5p inhibits FLSs proliferation and proinflammatory cytokine production by targeting XBP1, a key regulator of ER stress. However, the precise mechanism by which ER stress is involved in miR-34a-5p’s regulation remains incompletely understood [38]. Our study is the first to reveal the complete regulatory loop of miRNAs in the context of ER stress, encompassing downstream angiogenesis and osteoclastogenesis processes. This provides a more comprehensive understanding of miRNA regulation in RA, particularly in an ER stress-dependent manner.

Crebrf, also known as LRF, serves as a specific negative regulator of Creb3 (cAMP-responsive element-binding protein 3). Creb3, alternatively known as LUMAN, is a crucial member of the Creb family. It is a membrane-bound transcription factor that plays a significant role in the unfolded protein response (UPR) during ER stress. Creb3 plays a vital role in reducing the workload of the ER by decreasing protein translation [39]. The involvement of Creb3 in ER function is multifaceted, as it mediates communication between ATF6 and XBP1 while also regulating calcium homeostasis and ATP production [40]. Crebrf restricts the nuclear translocation of Creb3, promotes Creb3 degradation, and thus regulates downstream UPR elements. In mouse embryonic fibroblast cells, Crebrf could sequester nuclear CREB3 out of the nucleus and promote CREB3 protein degradation. Crebrf knockdown significantly elevates the expression of ER stress response markers, indicating that Crebrf acts as a negative regulator of UPR and ER stress [41]. In our study, Crebrf was identified as the direct downstream target of miR-378 in the regulation of ER stress response in RA-FLSs, which implies a significant role for Crebrf in regulating ER stress in the pathogenesis of RA. CREBRF expression in PBMCs was discovered to be downregulated in polyarticular juvenile idiopathic arthritis, as well as autoimmune uveitis [42], which further demonstrated its clinical and therapeutic significance in autoimmune diseases.

It is evident that miRNAs play a significant role in the progression of RA. The development of RA involves a complex network of cytokines, including IL-1, IL-6, IL-10, IL-17, IL-18, IL-21, and TNFα, which govern immune and inflammatory processes. These cytokines are effectively regulated by miRNAs, exerting a substantial influence on the various stages of RA, including the early, acute, and chronic phases [43]. In RA patients, elevated expression of miR-146, miR-155, miR-132, and miR-16 has been observed in peripheral blood mononuclear cells (PBMCs). MiR-146 and miR-155 are closely linked to RA as they control the molecular mechanisms of cytokines and cellular behaviors [44–46]. Specifically, in preclinical research, attempts have been made for several years to apply miRNA mimics or viral delivery systems to animal models for RA treatment. Yang et al. were the first to demonstrate that intra-articular injection of miR-30a could significantly rescue the inflammatory joint destruction in RA mice [47]. Kong et al. also demonstrated that miR-20a mimics injection impeded osteoclast proliferation and differentiation and thus improved bone erosion in a CIA mouse model [48]. Notably, a single ankle joint injection of pre-miR-124 could spread into distant joints rather than accumulate at the injection site, leading to reduced arthritic tissue destruction in multiple joints [49]. The tail vein injection of miR-320a-mediated exosomes also alleviated arthritis severity in CIA mice [50]. These findings further support the therapeutic potential of tail vein injection of lentiviral-mediated anti-miR-378 in ameliorating joint inflammation, blood vessel formation, and bone destruction throughout the body.

In conclusion, this study demonstrates that miRNA-378^high^ mice develop severe arthritis after collagen injection compared to WT mice. Overexpression of miR-378 was found to activate the ER stress response, subsequently promoting angiogenesis and osteoclastogenesis in RA tissues. Exploring the potential of developing miRNA-378 inhibitors as novel diagnostic and treatment tools for RA is warranted.

## Supplementary information

Supplementary data

## Data Availability

Raw data were generated at the Chinese Univeristy of Hong Kong. Derived data supporting the findings of this study are available from the corresponding author GL on request.
