# Supplementary material for: MiR-378 exaggerates angiogenesis and bone erosion in collagen-induced arthritis mice by regulating endoplasmic reticulum stress
Source: Cell Death Dis. 2024 Dec 18;15(12):910. doi: 10.1038/s41419-024-07193-5 (PMC11655635; doi:10.1038/s41419-024-07193-5)
Supplement: Supplementary file 1 — Supplementary data [file 41419_2024_7193_MOESM1_ESM.docx]

**
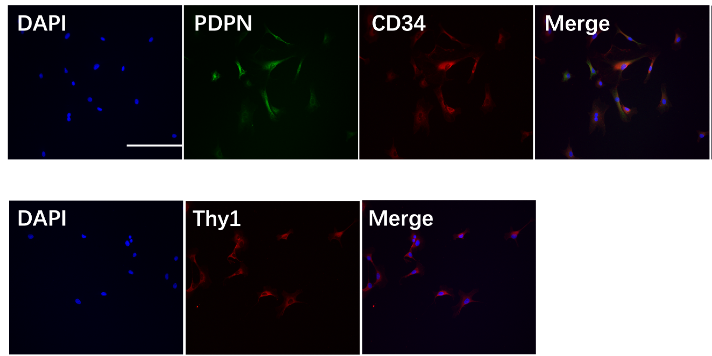
**

**Supplementary Figure 1.** Fibroblast-like synoviocytes (FLSs) were characterized by IF staining using PDPN, CD34 and Thy1antibodies. Scale bar: A, 100 μm.


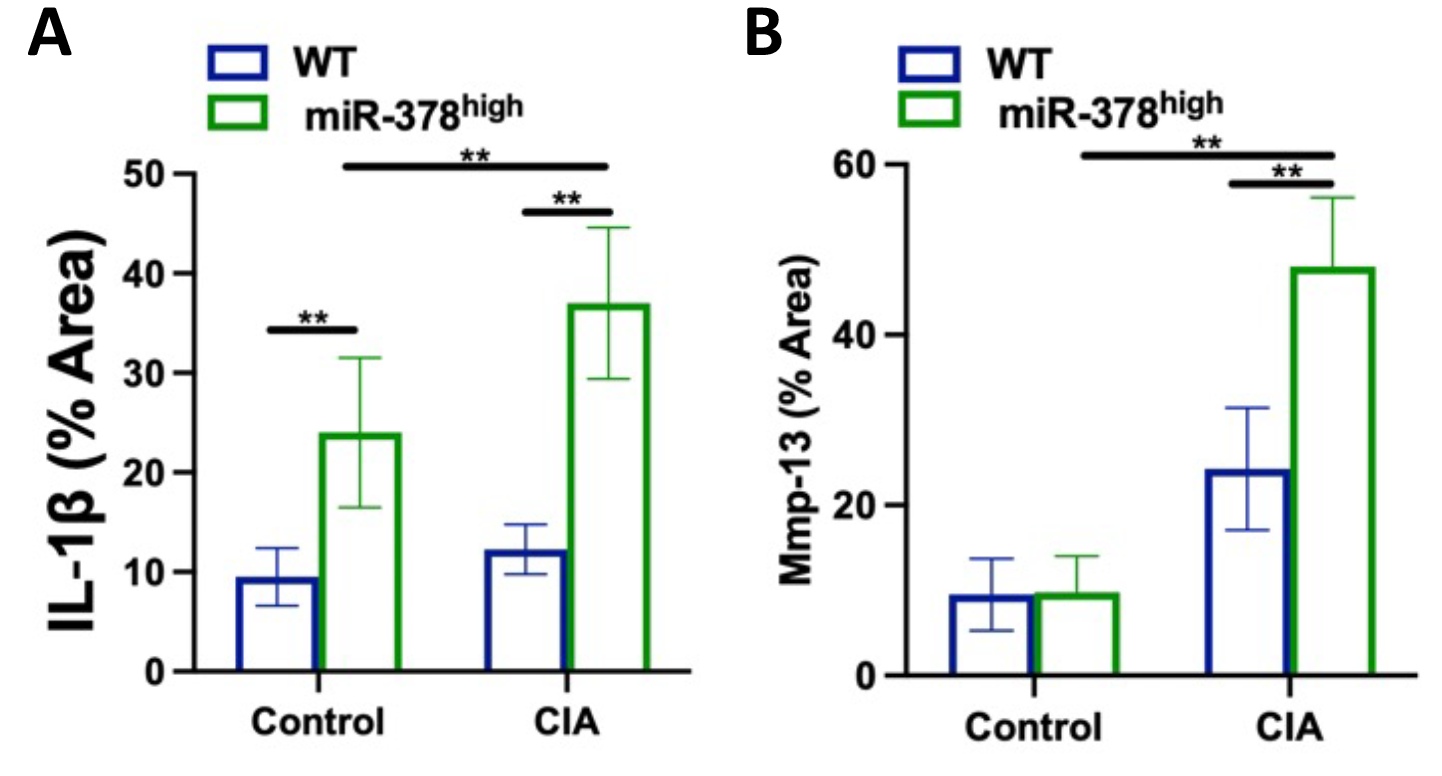


**Supplementary Figure 2.** Semi-quantitative analysis of the percentage of IL-1β (A) and Mmp13 (B) positive areas in ankle from two group of mice at week 5 after CIA induction (n=6; **p<0.01).


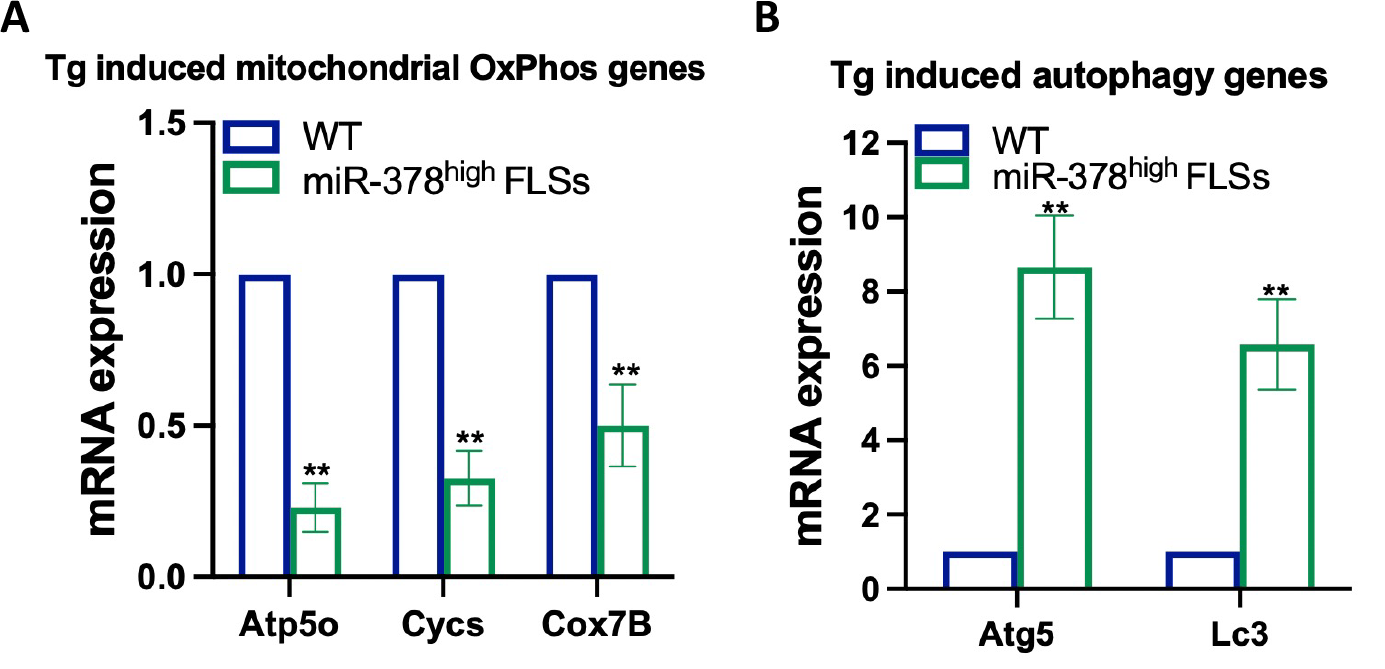


**Supplementary Figure 3. (A)** Real-time PCR analysis of the mRNA expression level of mitochondrial OxPhos genes in WT and miR-378^high^ FLSs upon Tg induction, including Atp5o, Cycs and Cox7B. **(B)** Real-time PCR analysis of the mRNA expression level of autophagy related genes in WT and miR-378^high^ FLSs upon Tg induction, including Atg5 and LC3 (n=6; **p<0.01).


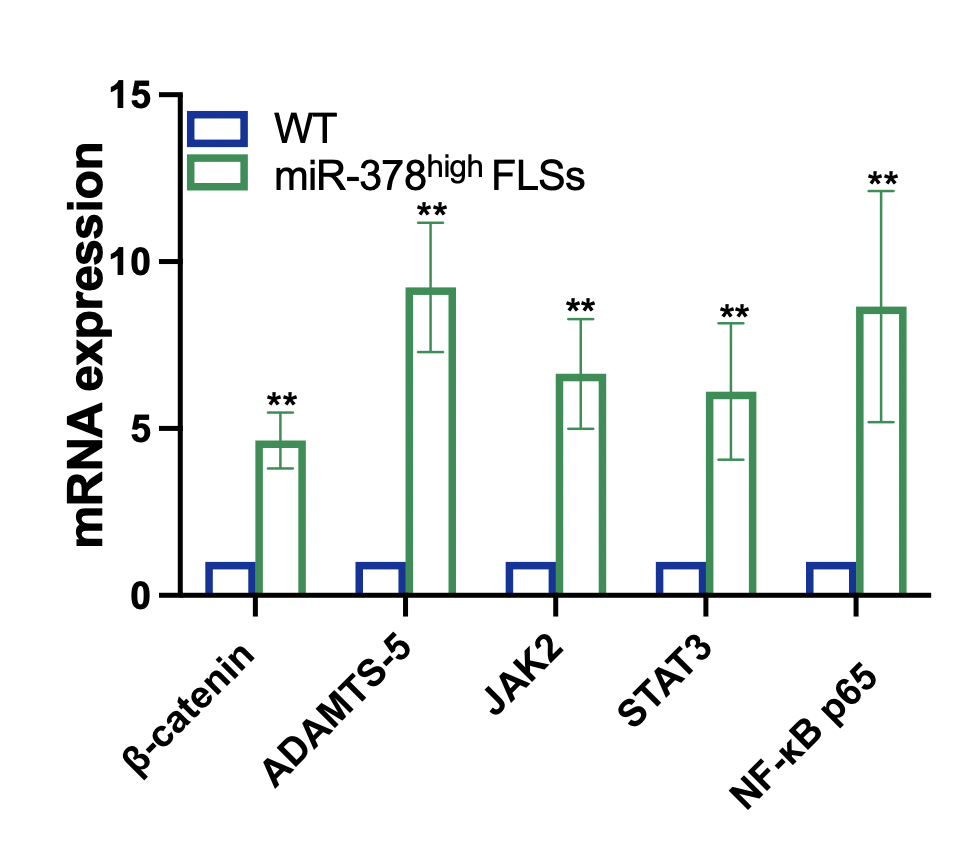


**Supplementary Figure 4.** Real-time PCR analysis of the mRNA expression level of inflammatory response related genes in WT and miR-378^high^ FLSs, including β-catenin, ADAMTS-5, JAK2, STAT3 and NF-κB p65 (n=6; **p<0.01).

**Supplementary Figure 5.** miR-378 expression level in isolated FLS of WT and miR-378^high^ mice. The relative miRNA expression was normalized to mmu-miR-103a-3p (n=6; ***p<0.001).

**
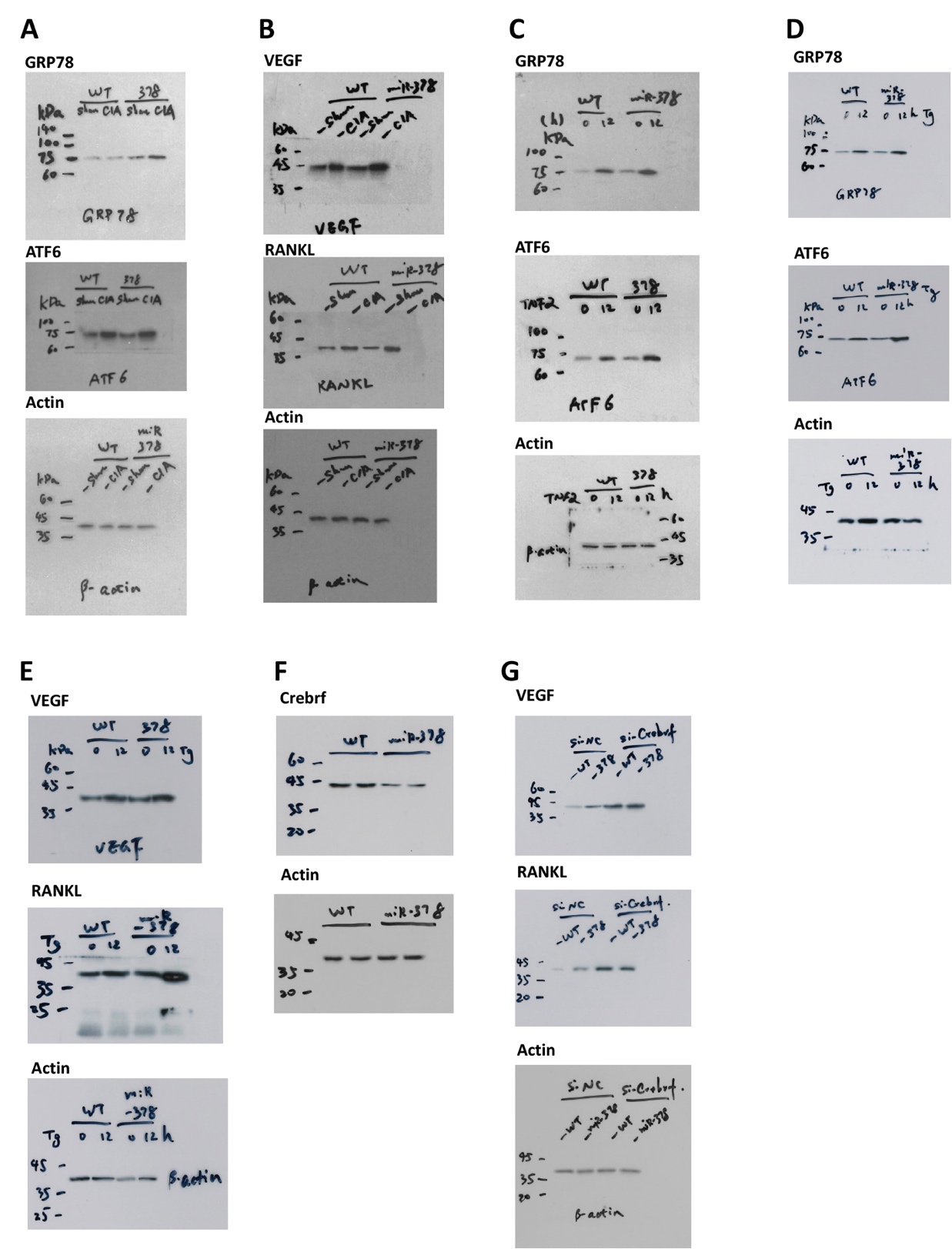
**

**Supplementary Figure 6**. **A-G.** Full length uncropped original Western blots used in Figure 2C (A), 2H (B), 3B (C), 3E (D), 4B (E), 5D (F) and 5G (G).

**Supplementary Table 1. Primers used for real-time PCR analysis**

| **Primers for Real-time PCR** | | | | |
| --- | --- | --- | --- | --- |
| Gene ID | Accession No. | F/R | Sequence | Product size (bp) |
| m-Atf6 | NM_001081304 | F  R | AATTCTCAGCTGATGGCTGT  TGGAGGATCCTGGTGTCCAT | 323 |
| m-Grp78 | NM_001163434 | F  R | GTGTTCAAGAACGGCCGCGTG  GTTTGCCCACCTCCAATATCAAC | 281 |
| m-Chop | NM_007837 | F  R | AAGATGAGCGGGTGGCAGCG  GCACGTGGACCAGGTTCTGCT | 121 |
| m-Xbp1 | NM_013842 | F  R | GAACCAGGAGTTAAGAACACG  AGGCAACAGTGTCAGAGTCC | 205 |
| m-Vegf | NM_001025250 | F  R | ATGGATGTCTACCAGCGAA  CTGCTGTGCTGTAGGAAGCTC | 251 |
| m-eNos | NM_008713 | F  R | GACCCTCACCGCTACAACAT  GCTCATTTTCCAGGTGCTTC | 199 |
| m-bFgf | NM_008006 | F  R | AGCGACCCACACGTCAAACT  CGTCCATCTTCCTTCATAGCAAG | 104 |
| m-Trap | NM_001102405 | F  R | CTGGAGTGCACGATGCCAGCGACA  TCCGTGCTCGGCGATGGACCAGA | 419 |
| m-Rankl | NM_011613 | F  R | AGCCGAGACTACGGCAAGTA  AAAGTACAGGAACAGAGCGATG | 201 |
| m-NFATc1 | NM_016791 | F  R | CCGTTGCTTCCAGAAAATAACA  TGTGGGATGTGAACTCGGAA | 152 |
| mGAPDH | NM_001289726 | F  R | CGTCCCGTAGACAAAATGGT  TTGATGGCAACAATCTCCAC | 110 |

“m” stands for “mouse”.
